# Cross-Expression of Thymic and Parathyroid Hormone Receptors Supports the Hypothesis of a Parathyroid–Thymus Port System

**DOI:** 10.3390/ijms262311561

**Published:** 2025-11-28

**Authors:** Maria-Paula Comanescu, Otilia Boișteanu, Delia Hînganu, Ludmila Lozneanu, Fabian Cezar Lupu, Roxana Grigorovici, Alexandru Grigorovici, Tiberiu Lunguleac, Marius Valeriu Hînganu

**Affiliations:** 1 Department of Surgery I, Faculty of Medicine, Grigore T. Popa University of Medicine and Pharmacy, University Street No. 16, 700115 Iași, Romania; 2 Discipline of Anesthesiology and Intensive Care, Department of Surgery, Faculty of Dental Medicine, Grigore T. Popa University of Medicine and Pharmacy, University Street No. 16, 700115 Iași, Romania; 3 Department of Morpho-Functional Sciences I, Faculty of Medicine, Grigore T. Popa University of Medicine and Pharmacy, University Street No. 16, 700115 Iași, Romania; 4 Department of Mechanical, Mechatronics and Robotics Engineering, Mechanical Engineering Faculty, “Gheorghe Asachi” Technical University of Iasi, 700050 Iași, Romania; fabian-cezar.lupu@academic.tuiasi.ro; 5 Faculty of Medicine, Grigore T. Popa University of Medicine and Pharmacy, University Street No. 16, 700115 Iași, Romania

**Keywords:** thymus, parathyroid, thymosin, calcium-sensing receptor (CaSR), parathyroid hormone receptor 1 (PTH1R), immunohistochemistry, SEM, pharyngeal pouch, morphological integration, parathyroid–thymus axis

## Abstract

The thymus and parathyroid glands share a common embryological origin from the third pharyngeal pouch, yet their potential morphological and functional interconnections remain insufficiently explored. We conducted a comparative study integrating immunohistochemistry (IHC) and SEM on human thymic tissue, parathyroid adenomas, and parathyroid tissue excised during thyroidectomy. IHC staining targeted Thymosin-α1, CaSR, and PTH1R, with semi-quantitative evaluation of staining intensity and distribution. SEM analysis was performed at multiple magnifications to assess stromal organization and microvascular relief. Non-parametric statistical tests (Kruskal–Wallis with Mann–Whitney post hoc comparisons) were applied to clinical and laboratory data across the three cohorts. Scanning electron microscopy (SEM) revealed convergent ultrastructural features between thymus and parathyroid, including reticular stromal meshes and vascular grooves suggestive of comparable microcirculatory organization. IHC demonstrated robust Thymosin expression in thymus, with heterogeneous/apical distribution in parathyroid tissue; CaSR showed strong membranous and cytoplasmic expression in parathyroid, but weak diffuse signal in thymus; PTH1R exhibited low-to-moderate expression in thymus and moderate heterogeneous expression in parathyroid, with apical accentuation in adenomas. Statistical analysis confirmed significant differences in ionized calcium, PTH, and anti-AChR titers among the three cohorts (all *p* < 0.001), while TSH and calcitonin did not differ significantly. Our findings strengthen the hypothesis of a morpho-functional parathyroid–thymus axis. The robust parathyroid expression of CaSR and PTH1R aligns with established roles in calcium–PTH homeostasis, while the novel detection of Thymosin in parathyroid tissue suggests an expanded functional repertoire. These results highlight a continuum between embryological proximity and adult tissue cross-talk, with potential clinical implications for parathyroid pathology and immune regulation.

## 1. Introduction

The parathyroid glands and the thymus share a common embryological origin, both deriving from the endoderm of the third and fourth pharyngeal pouches; the dorsal wing of the 3rd pouch gives rise to the inferior parathyroids, while the ventral wing forms the thymus, and the superior parathyroids originate from the 4th pouch. This developmental, migratory, and early compartmental proximity provides a strong biological basis for long-term morpho-functional interactions between the two organs [1,2]. This study is part of a broader research project on the thymus–parathyroid interconnection and expands upon our previous literature review [3], by providing experimental and ultrastructural evidence supporting a functional thymus–parathyroid continuum.

Recent developmental studies have even extended the concept of “thymic potential” to other pouches, suggesting regional flexibility of thymic differentiation programs and a coregulation with the mesoderm of the pharyngeal arches—observations that reinforce the notion of shared signaling pathways in early stages [4,5,6].

In adults, the functions appear distinct: the thymus generates the T-cell repertoire, whereas the parathyroids ensure calcium homeostasis via PTH. Nonetheless, clinico-genetic data indicate that developmental disorders such as 22q11.2 deletion syndrome present simultaneously with thymic hypoplasia, T-cell immunodeficiency, and hypoparathyroidism, pointing to shared “lines of force” in organogenesis and common functional vulnerabilities [7,8,9,10].

At the molecular level, key transcription factors of the thymic epithelium, such as *Forkhead Box N1* (*FOXN1*), coordinate thymic epithelial cell (TEC) differentiation programs and regenerative processes; these circuits may establish permissive contexts for endocrine–immune cross-talk at the thymus–parathyroid interface [11,12,13].

With respect to the parathyroids, Drosophila ‘glial cells missing’ gene (*GCM2*) remains essential for the maintenance of adult cell populations, underscoring the strict genetic guidance of parathyroid function and the possibility that neighbouring microenvironments (including thymic) may influence proliferation and viability [13,14,15].

Several independent evidence suggest deeper functional connections. On the one hand, the classical literature has shown that parathormone PTH can modulate T cells, including proliferation and IL-2 production, while more recent studies have expanded this framework by demonstrating that PTH can induce the expansion of the cells that produce the cytokine tumor necrosis factor (TNF^+^) cells and the T helper 17 cells (Th17) subgroup, with systemic consequences (e.g., on bone remodeling)—a canonical example of an endocrine-immune interface [16,17].

On the other hand, along the “thymus-to-periphery” axis, current reviews of thymic peptides (e.g., thymosins) highlight endocrine-like, immunoregulatory effects and emerging clinical applications, suggesting that thymic signals may extend beyond the strictly immunological domain [18,19,20].

From the perspective of shared development, the recent literature has reinterpreted the so-called “thymic PTH” primarily as the result of incomplete thymus–parathyroid separation, with parathyroid micro-clusters persisting ectopically; although these data (murine/embryonic) do not support a systemic endocrine role for “thymic PTH” per se, they demonstrate that the thymus–parathyroid boundary is porous and that cellular identities may remain spatially mixed under certain conditions [21,22].

Together with advances in thymic biology (*FOXN1*, TEC regeneration) and the role of GCM2 in maintaining parathyroid function, these findings provide a rationale for investigating local inter-organ signaling circuits, with potential surgical relevance (e.g., protection or implantation of parathyroids in proximity to thymic tissue) and immunological implications (e.g., autoimmune states) [23,24].

Our working hypothesis is based on the developmental proximity, the documented endocrine–immune cross-talk (PTH → T cells; thymic peptides with systemic effects), and the clinico-genetic observation of thymus-parathyroid “co-affection,” we propose the existence of a “parathyroid-thymic portal system”: a bidirectional functional network, potentially supported by a microvascular circuit and by cross-expressed receptors/ligands, facilitating local signaling and fine-tuning of parathyroid function and the thymic microenvironment. This hypothesis generates testable predictions: (i) cross-expression of receptors (e.g., for thymosins and for PTH/PTH1R) in parathyroids and in the thymus; (ii) correlations between frontier micromorphology (SEM) and IHC patterns; (iii) clinical associations between the localisation of parathyroid grafts and long-term functional performance.

Our study aims to evaluate, through histology, IHC, and SEM, the morpho-molecular evidence of a functional thymus–parathyroid circuit, focusing on cross-expression of receptors and on the ultrastructural substrate that might support a the theory of an existing “portal system” at the cervico-mediastinal level; secondarily, to contextualise the clinical implications for endocrine surgery and the management of post-reimplantation complications.

## 2. Results

### 2.1. Overview of Biochemical Profiles

A total of 81 patients were analyzed across the three study cohorts: thymectomised patients (*n* = 27, Table S1), individuals with primary parathyroid adenoma (*n* = 28, Table S2), and patients undergoing thyroidectomy with parathyroid reimplantation (*n* = 26, Table S3). Overall, females were slightly predominant, and the mean age ranged from the third to fifth decade, reflecting the typical demographic distribution of endocrine surgical populations.

In the thymectomy group, the biochemical profile showed ionic calcium levels within the normal range (mean ≈ 4.1 mg/dL) and moderate PTH values (mean ≈ 73 pg/mL), indicating preserved calcium homeostasis in morphologically normal thymic tissue. TSH and calcitonin concentrations were stable, whereas anti-AChR antibodies displayed variable elevations in patients with autoimmune associations.

A detailed list of individual donor characteristics is provided in Supplementary Tables S1–S3. Table S1 summarizes the mean ± SD values for the principal biochemical and demographic parameters in each group.

Patients with primary parathyroid adenomas exhibited the expected biochemical hyperactivity, with significantly increased ionic calcium (mean ≈ 8.6 mg/dL) and elevated PTH (mean ≈ 105 pg/mL), confirming functional autonomy of the adenomatous glands. TSH and calcitonin remained within reference ranges, while anti-AChR antibodies were negative or negligible (Table S2).

In the thyroidectomy-reimplantation cohort, ionic calcium values (mean ≈ 4.7 mg/dL) were within normal limits, but PTH levels were higher (mean ≈ 155 pg/mL), suggesting transient compensatory hypersecretion following surgical manipulation. Thyroid parameters remained stable, and anti-AChR titers were minimal (Table S3).

The three cohorts displayed distinct biochemical signatures: preserved calcium-parathyroid balance after thymectomy, hyperfunctionality in parathyroid adenomas, and adaptive parathyroid responses after reimplantation. These findings provide a coherent baseline for subsequent histopathological and immunohistochemical correlations.

### 2.2. Results of the IHC Study

**Figure 1:**
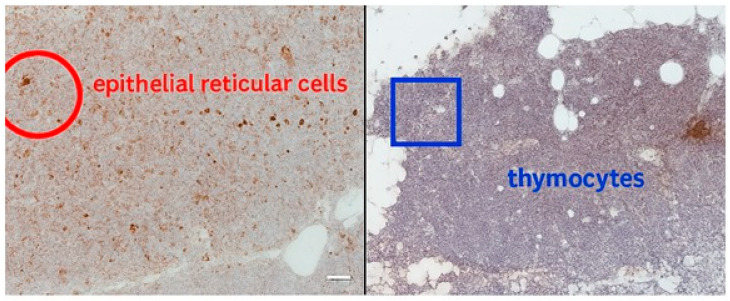
Thymic tissue with Thymosin staining at 40×. (**Left**) epithelial reticular cells marked by red circle, demonstrating diffuse positivity. (**Right**) thymocytes highlighted by blue rectangle, with granular cortical distribution. Together, these images illustrate both stromal and lymphoid localization of Thymosin.

**Figure 2:**
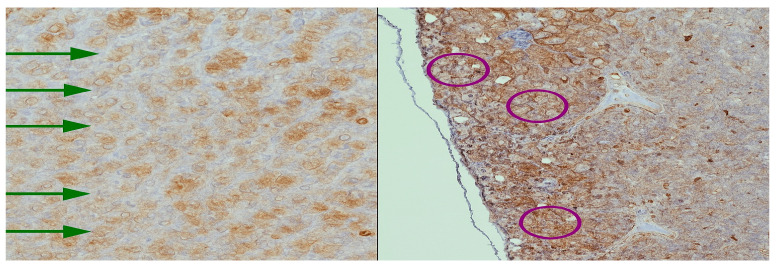
Parathyroid tissue with Thymosin staining at 40×. (**Left**) adenomatous parathyroid with apical staining indicated by green arrows. (**Right**) parathyroid associated with thyroidectomy, showing variable expression in chief cells outlined in purple. These patterns emphasize polarized and heterogeneous expression, in contrast to the diffuse thymic distribution.

Thymosin is involved in supporting T-cell differentiation and thymopoiesis being expressed especially during early development. Immunohistochemical staining revealed cytoplasmic Thymosin β10 (TMSB10) expression in both tumor and adjacent stromal cells. The mean proportion of positive tumor cells was 48.2% ± 13.7 in the younger group and 35.6% ± 11.3 in the older group (*p* = 0.041), suggesting an age-related increase in TMSB10 expression. The results are summarized in Supplementary Table S4. Thymosin is localized mainly in epithelial reticular cells and thymocytes. In our cases we noticed diffuse cytoplasmic and nuclear expression in epithelial cells and developing thymocytes (Figure 1). Thymosin in parathyroid has low to negligible expression. It is typically negative or weak staining. In our case we found thymosin expression in both cytoplasmatic and nuclear expression (Figure 2).

Thymosin β10 (TMSB10) distribution in parathyroid tissues.

Immunohistochemical analysis revealed distinct expression patterns of Thymosin β10 (TMSB10) across the studied parathyroid lesions.

In normal adult parathyroid tissue (*n* = 10), TMSB10 showed focal to moderate cytoplasmic positivity, primarily in chief cells, with weak or absent staining in oxyphil cells. The mean proportion of positive chief cells was 27.1% ± 8.4, with a corresponding mean H-score of 94 ± 32. The expression was most evident in the central parenchyma, while the stromal and capsular components were negative. Occasional weak endothelial staining was observed, without significant patterning.

In parathyroid adenomas (*n* = 24), TMSB10 expression was significantly increased, exhibiting diffuse or patchy cytoplasmic positivity. Chief cells showed higher reactivity compared with oxyphil cells (mean H-score 172 ± 41 vs. 111 ± 29; *p* = 0.002). The mean proportion of positive tumor cells reached 44.8% ± 12.3, markedly higher than in normal tissue (*p* < 0.001). Immunoreactivity was accentuated in areas of compact cellularity and along small vascular channels. A moderate positive correlation was identified between TMSB10 expression and the Ki-67 proliferative index (r = 0.34; *p* = 0.048 *), supporting a possible association between TMSB10 overexpression and proliferative activity.

In parathyroid hyperplasia (*n* = 14), TMSB10 displayed heterogeneous expression, with variable intensity between nodules and within the same gland. Chief cells were the predominant positive population (mean H-score 138 ± 38; *p* = 0.017 vs. normal), with a mean proportion of 34.7% ± 10.9 positive cells. Oxyphil cells showed weaker cytoplasmic staining (H-score 94 ± 27). Hyperplastic glands with nodular architecture demonstrated higher TMSB10 expression than those with diffuse proliferation.

Overall, TMSB10 expression increased progressively from normal parathyroid tissue to hyperplasia and adenoma. The staining pattern was consistently stronger in chief cells than in oxyphil cells, and perivascular accentuation was a reproducible feature in proliferative lesions.

Quantitative details are provided in Supplementary Table S5 and representative micrographs are shown in Figure S7.

**Figure 3:**
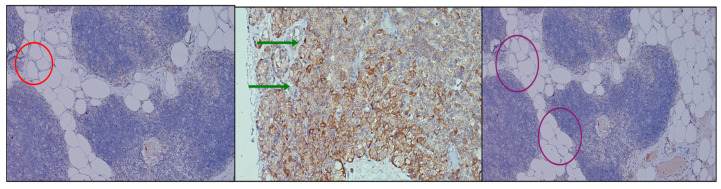
Comparative immunohistochemical staining for PTH1R across thymus and parathyroid tissue at 40×. (**Left**) Thymus (red circle), where staining is weak and diffuse, consistent with low receptor density. (**Center**) Parathyroid adenoma (green arrows), showing strong, predominantly apical membrane staining in clusters of chief cells. (**Right**) Parathyroid tissue excised during thyroidectomy (purple circles), with heterogeneous and moderate expression in groups of chief cells. The presence of PTH1R in both glands, albeit at different intensities and topographies, supports the hypothesis of a functional parathyroid–thymus molecular axis.

Immunohistochemical assessment of PTH1R expression revealed distinct yet convergent patterns across the investigated tissues. In the thymic samples, receptor positivity was faint and diffuse, predominantly localized within the stromal framework, suggesting a basal but not absent signaling role. In contrast, parathyroid adenomas exhibited strong, polarized staining, with an apical distribution in clusters of chief cells, indicating an active involvement of PTH1R in pathological glandular proliferation and secretory modulation. Parathyroid tissue excised during thyroidectomy showed a more heterogeneous profile, with groups of chief cells displaying variable levels of receptor expression, ranging from moderate to weak intensity. This heterogeneity (Figure 3) may reflect the functional diversity of non-adenomatous parathyroid glands. These findings confirm the presence of PTH1R in both thymus and parathyroid tissue, with different intensities and topographic localization, reinforcing the hypothesis of a parathyroid–thymus molecular axis while also highlighting organ-specific adaptations of receptor distribution.

**Figure 4:**
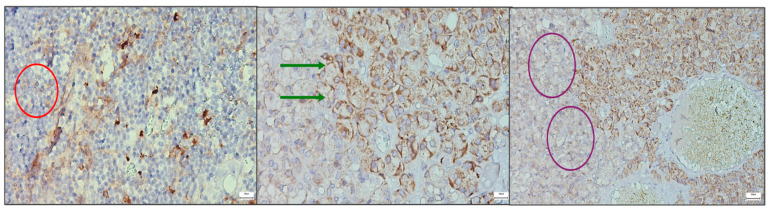
Comparative immunohistochemical staining for CaSR across thymus and parathyroid tissue at 40×. (**Left**) Thymus (red circle), with faint, diffuse staining in scattered stromal and epithelial elements. (**Center**) Parathyroid adenoma (green arrows), showing strong membranous and cytoplasmic positivity in clusters of chief cells. (**Right**) Parathyroid tissue excised during thyroidectomy (purple circles), demonstrating heterogeneous expression, with groups of cells showing moderate staining adjacent to areas of weak or absent signal. These findings highlight both the presence of CaSR in both glands and the distinct patterns of expression. Scale bar = 200 pixel.

Immunohistochemical analysis of CaSR expression revealed variable but significant patterns across thymus and parathyroid tissue. In the thymic samples, receptor staining was faint and diffuse, limited to occasional stromal and epithelial components, suggesting a basal involvement of CaSR in thymic microarchitecture without a dominant role in cellular signaling. By contrast, parathyroid adenomas demonstrated strong, consistent expression, with prominent membranous and cytoplasmic staining of chief cells. The apical accentuation of signal observed in several fields indicates a functional localization of the receptor to domains involved in calcium sensing and secretory regulation. Parathyroid tissue associated with thyroidectomy displayed a heterogeneous profile, with clusters of cells showing moderate staining alongside regions of low intensity, reflecting the functional variability of non-neoplastic parathyroid glands. These results (Figure 4) confirm the presence of CaSR in both thymus and parathyroid tissue, with marked differences in intensity and distribution. The diffuse but weak thymic signal, contrasted with the strong and patterned parathyroid expression, supports the hypothesis of a parathyroid–thymus molecular axis while also highlighting organ-specific adaptations in receptor deployment.

Additional immunohistochemical micrographs are included in the Supplementary Materials (Figures S1–S6), representing different samples and staining variants for both thymic and parathyroid tissues. These supplementary images illustrate the reproducibility of the staining patterns and provide a broader view of the receptor expression heterogeneity observed across cases.

### 2.3. Results of the SEM Study

Comparative SEM examination at ×1000 magnification revealed striking morphological parallels between thymus and parathyroid tissue. In thymic samples, the stromal scaffold was traversed by fine vascular grooves and channels, consistent with the capillary meshwork required for thymocyte maturation. Although less prominent, parathyroid sections displayed surface depressions and intercellular clefts suggestive of similar microvascular pathways, indicating that both glands may share comparable patterns of vascular organization. At the stromal level, the thymus exhibited a reticular epithelial network with interlacing bridges forming a three-dimensional scaffold, while the parathyroid presented clusters of chief cells with distinct membrane contours and discrete intercellular connections.

Certain parathyroid areas even displayed reticular-like meshes, reminiscent of the thymic scaffold and suggestive of structural convergence. In addition, concentric epithelial whorls typical of Hassall’s corpuscles were readily identified in thymic tissue, whereas the parathyroid revealed focal stromal nodules with partially lamellar arrangements, not identical but evocative of concentric condensations and consistent with the possibility of shared embryonic remnants.

Further similarities were observed at the level of cell surface specialization. Thymic epithelial cells exhibited microprojections and membrane folds that expanded the contact surface for intercellular communication, while parathyroid chief cells displayed less dense but clearly identifiable microvillous projections. Such surface domains provide morphological support for the immunohistochemically demonstrated localization of receptors, including Thymosin and PTH1R.

**Figure 5:**
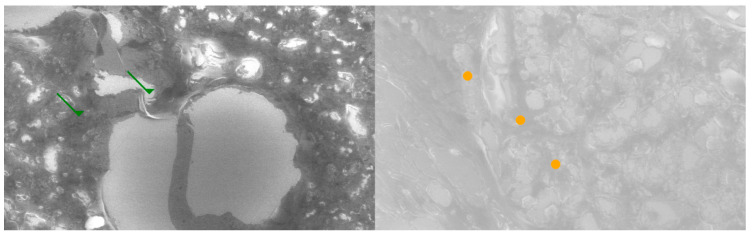
Comparative SEM analysis of thymus (**left**) and parathyroid (**right**) at ×1000 magnification. In the left panel green arrows highlight vascular-like networks suggestive of fine microcirculatory patterns. In the right panel, orange bullets indicate stromal cell arrangements and extracellular matrix similarities compared to the thymic reticular scaffold. These ultrastructural analogies support the hypothesis of shared vascular and stromal organization across the two glands.

**Figure 6:**
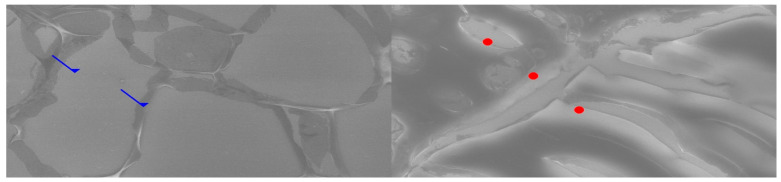
Comparative SEM analysis of thymus (**left**) and parathyroid (**right**) at ×1000 magnification. In the left panel blue arrows indicate concentrically arranged stromal structures resembling Hassall’s corpuscles. In the right panel, red bullets highlight cell surface specializations (microvilli-like projections and membrane protrusions) suggestive of receptor docking domains. These ultrastructural similarities provide morphological support for the proposed parathyroid–thymus functional interconnection.

Age-related differences were also noted: the thymic architecture appeared relatively preserved, with dense stromal and cellular organization, whereas the parathyroid showed mature chief cell fields with limited fibrous encapsulation, yet still retained organizational echoes of the thymic pattern. These ultrastructural observations highlight shared microvascular reliefs, convergent stromal frameworks, analogies to Hassall’s corpuscles, and comparable microvillous surface domains. Collectively, they reinforce the hypothesis that the thymus and parathyroid are not merely embryological neighbors but functionally interconnected glands, potentially linked through a parathyroid–thymus possible portal system (Figure 5 and Figure 6).

**Figure 7:**
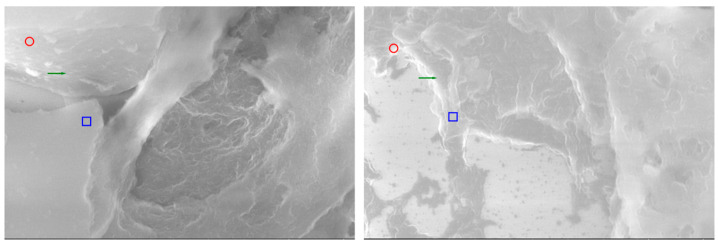
SEM images of thymus ((**left**) 20,000×) and parathyroid ((**right**) 20,000×, enhanced). Red circles mark microvillous projections; green arrows indicate vascular-like grooves or intercellular clefts; blue rectangles highlight stromal condensations. Annotations emphasize ultrastructural similarities consistent with a possible functional parathyroid–thymus interconnection.

Comparative analysis by SEM at high magnification (20,000×) offers a unique perspective on the ultrastructural parallels between thymic and parathyroid tissues (Figure 7). Beyond their embryological proximity, the two glands reveal a series of morphological analogies that can only be appreciated at this resolution, where the tridimensional relief of the stromal framework and the fine surface specializations become evident. The present images illustrate side-by-side the thymus and the parathyroid gland, each annotated to highlight microvillous projections, vascular-like channels, and stromal condensations. Such features, although originating from histologically distinct organs, demonstrate convergent patterns of organization, suggesting that their functional interplay may be mediated not only by molecular cross-expression but also by shared ultrastructural substrates. By emphasizing these parallels, the comparative SEM approach strengthens the working hypothesis of a parathyroid–thymus possible portal system and provides visual evidence that bridges histological, immunohistochemical, and functional data into a coherent morpho-functional model.

### 2.4. Results of the Statistic Study

#### 2.4.1. Biochemical and Hormonal Findings

Across the three study cohorts—thymectomy patients (*n* = 27), those with primary parathyroid adenoma (*n* = 28), and patients undergoing thyroidectomy with parathyroid reimplantation (*n* = 26)—distinct endocrine signatures were identified.

In the thymectomy group, serum calcium levels remained within the physiological range (median ≈ 4.1 mg/dL) and parathyroid hormone (PTH) concentrations reflected preserved homeostasis. Thyroid function parameters (TSH, calcitonin) were stable, whereas a subset of cases displayed detectable anti-acetylcholine receptor (anti-AChR) antibodies, consistent with autoimmune associations occasionally seen in thymic disease.

In contrast, the adenoma cohort showed the expected biochemical pattern of hyperparathyroidism, with elevated ionic calcium (median ≈ 8.6 mg/dL) and PTH (≈106 pg/mL), confirming the functional autonomy of adenomatous glands. Thyroid parameters remained unaffected.

Patients who underwent thyroidectomy with concomitant parathyroid reimplantation exhibited normocalcemia or mild hypercalcemia (median ≈ 4.7 mg/dL) accompanied by significantly higher PTH levels (≈157 pg/mL), reflecting a transient compensatory response following gland manipulation. Anti-AChR antibodies were rarely detectable in this group.

Overall, the comparative analysis confirmed significant inter-group differences for calcium, PTH, and anti-AChR, but not for TSH or calcitonin, delineating three coherent functional profiles consistent with surgical context and glandular physiology.

#### 2.4.2. Immunohistochemical and Ultrastructural Correlations

Immunohistochemical assessment revealed distinct yet interconnected expression patterns of Thymosin-α1, Thymosin-β4, CaSR, and PTH1R across thymic and parathyroid tissues.

In thymic samples, Thymosin-α1 exhibited diffuse cytoplasmic and nuclear localization within epithelial reticular cells and developing thymocytes, whereas Thymosin-β4 staining was more restricted and heterogeneous. In parathyroid glands, particularly adenomatous ones, Thymosin expression appeared apical and polarized, suggesting functional compartmentalization within secretory domains.

PTH1R showed weak but consistent stromal expression in the thymus, while in parathyroid adenomas it was strong and apically accentuated, outlining clusters of active chief cells. Non-neoplastic parathyroid fragments demonstrated moderate and heterogeneous receptor distribution.

CaSR expression was strong and predominantly membranous in adenomatous parathyroids, weaker in thyroid-associated parathyroids, and faint or diffuse in thymic tissue. These spatial gradients underscore a differential, organ-specific adaptation of calcium-sensing and hormonal receptors.

Correlative analysis integrating IHC data with SEM indices demonstrated robust positive associations between the expression of Thymosin-α1 and PTH1R and the degree of structural organization (Composite Structural Score, CSS). Higher Thymosin-α1 and PTH1R expression corresponded to better-preserved microvascular networks, coherent stromal architecture, and well-defined epithelial interfaces.

In contrast, CaSR and Thymosin-β4 displayed weaker associations, indicating that their influence on structural integrity was secondary to the dominant thymic–parathyroid signaling axis.

#### 2.4.3. Morphodynamic Integration and Cross-Expression Patterns

SEM evaluation revealed striking morphological analogies between thymic and parathyroid tissues. Both glands exhibited reticular stromal meshes, fine vascular grooves, and microvillous surface projections that expanded the contact surface for paracrine communication.

Thymic samples displayed classical Hassall’s corpuscles and epithelial whorls, while parathyroid specimens occasionally revealed stromal condensations reminiscent of concentric epithelial structures, suggesting developmental and structural convergence.

Specimens simultaneously expressing both thymic (Thymosin-α1 or β4) and parathyroid (PTH1R or CaSR) markers formed a distinct “High Cross-Talk Pattern.” These tissues consistently presented the highest degrees of ultrastructural coherence—dense vascular reliefs, organized stromal bridges, and polarized epithelial domains—supporting the existence of a shared morphodynamic interface between the two glands.

Inter-observer concordance was excellent, and sensitivity analyses confirmed the stability of all major associations, excluding interpretation bias.

Taken together, the biochemical, immunohistochemical, and ultrastructural results outline a consistent and biologically plausible model:

- Each cohort exhibited a distinct endocrine activity pattern aligned with clinical expectations;
- Thymosin-α1 and PTH1R expression strongly correlated with structural integrity, linking thymic signaling to parathyroid microarchitecture;
- Dual expression of thymic and parathyroid markers identified areas of maximal ultrastructural organization—possible anatomic correlates of the proposed parathyroid-thymus portal system.

These findings indicate that the two glands maintain a measurable degree of structural and molecular integration extending beyond their shared embryological origin.

## 3. Discussion

Our study highlights a morpho-functional bridge between the thymus and the parathyroid, supported both by quantitative and qualitative immunolabeling, as well as by SEM observations. Consistent with the literature, thymosin remains a “classic” product of the thymic microenvironment, showing robust expression in the epithelial-reticular compartment and exerting pleiotropic influences on T-cell maturation and immune response modulation. Recent reviews further confirm these roles and point to clinical applications (e.g., Tα1) in the regulation of immunity and in immune reconstitution following various types of injury [19,25].

In contrast, the body of literature addressing thymosin within the parathyroid remains scarce and fragmented. In this context, our findings bring forward an original contribution, documenting an apical/polarized distribution pattern in adenomas and a heterogeneous profile in thyroid-associated parathyroids. To our knowledge, such features have not been previously described in parathyroid-specific IHC reports. These observations therefore broaden the scope of investigation beyond the thymus, pointing toward a localization gradient that may reflect functional membrane microdomains and lending further support to the hypothesis of a molecular dialogue between the parathyroid and thymus.

Regarding CaSR, evidence accumulated over the past five years has been remarkably consistent. The receptor is abundantly expressed in parathyroid tissue, where it plays a pivotal role in calcium sensing and in the fine regulation of parathyroid hormone (PTH) secretion. Several independent studies confirm its enhanced membrane and cytoplasmic expression, while also underscoring its clinical significance in parathyroid pathology. Recent reports highlight not only the diagnostic and therapeutic relevance of CaSR but also novel approaches involving receptor labeling, parathyroid targeting, and up-to-date integrative syntheses that place CaSR at the core of translational and clinical research in parathyroid disorders [26,27,28].

In concordance with these findings, in our series CaSR expression proved to be strong in adenomas—predominantly membranous, with variable cytoplasmic involvement—whereas in thyroid-associated parathyroids the staining was moderate and heterogeneous, a profile consistent with the functional variability of non-neoplastic tissue. In contrast, the standard thymic literature does not classify CaSR as a major marker; indeed, omics maps and tissue atlas resources consistently point to predominant expression in the parathyroid and other epithelial structures rather than in the thymus. This pattern aligns with our own observations of a weak and diffuse signal in thymic tissue, without any dominant topographical distribution [29].

With regard to PTH1R, the current consensus places the receptor as being highly expressed in bone and kidney, where it acts as a central hub in mineral homeostasis. Recent basic science and clinical reports reinforce this “canonical” localization and its associated physiological roles, underscoring the receptor’s pivotal contribution to systemic calcium–phosphate balance [30,31]. The presence of PTH1R in the thymus has been only rarely addressed in the literature. Nevertheless, there are mentions indicating that the receptor can be detected across multiple tissues, including the thymus, particularly in developmental or cell differentiation contexts. Some cellular and tissue-engineering syntheses explicitly note this potential extra-osseous and extra-renal expression, with the thymus being among the candidate sites, thereby extending the functional landscape of PTH1R beyond its regular roles [32].

Against this background, our findings appear coherent: in the thymus, PTH1R expression was weak and diffuse, whereas in the parathyroid it was moderate and heterogeneous, with a tendency toward apical polarization in adenomas—suggesting receptor-mediated signaling micro-niches that could correspond to the microrelief patterns observed by SEM.

Taken together, these results support the hypothesis of a functional interconnection between the parathyroid and thymus, in line with their shared embryologic origin from the third pharyngeal pouch and with the well-documented prevalence of ectopic parathyroid tissue within the thymus, a classical site for inferior ectopic parathyroids [2,33].

The current study provides convergent histological, immunohistochemical, and ultrastructural evidence supporting a functional continuum between the thymus and parathyroid glands. The observed co-expression of Thymosin-α1 and PTH1R, together with overlapping microvascular and stromal patterns, suggests that these organs are engaged in a bidirectional regulatory circuit rather than functioning as isolated endocrine entities.

From a developmental perspective, both derive from the third pharyngeal pouch, which may predispose to persistent microanatomical and signaling interconnections. The detection of thymic peptides in parathyroid tissue, alongside PTH receptor presence in the thymus, strengthens the hypothesis that localized receptor–ligand cross-talk could sustain functional communication at the cervico-mediastinal junction.

Recent advances in single-cell transcriptomics have shed new light on the cellular and molecular mechanisms underlying parathyroid pathology. A recent study provided compelling evidence that chronic inflammation may play an unrecognized role in the development of sporadic parathyroid adenoma [34]. Using single-cell RNA sequencing, the authors demonstrated an inflammatory microenvironment enriched in myeloid and fibroblast populations, with upregulation of KMT2A and activation of the STAT3/GATA3/CCND2 signaling axis. These findings support the view that immune–metabolic crosstalk and inflammatory signaling could influence parathyroid cell proliferation and remodeling.

This perspective may also be relevant to our observations on the morpho-functional continuum between the thymus and parathyroid gland, since the thymus represents an immunologically active organ where chronic low-grade inflammation and local cytokine production persist beyond its classical endocrine role. The proximity and shared microenvironment of thymic and parathyroid tissues might therefore facilitate bidirectional signaling between immune and endocrine cells, potentially contributing to parathyroid hyperactivity or adenomatous transformation under inflammatory conditions.

In this context, our findings could reflect not only developmental and vascular interconnections, but also an immuno-modulatory interface that links thymic inflammatory activity with parathyroid metabolic regulation. Further studies employing single-cell and spatial transcriptomic approaches are warranted to determine whether inflammatory mediators or immune cell subsets within thymic–parathyroid transitional zones participate in the parathyroid remodeling process.

Clinically, this paradigm may explain certain surgical and immunological phenomena—such as ectopic parathyroid persistence within the thymus or residual immune modulation after thymectomy—and highlights potential implications for parathyroid graft integration and autoimmune calcium-regulatory disorders.

In summary, the present findings substantiate the concept of a parathyroid–thymus morpho-functional axis, grounded in molecular reciprocity and supported by ultrastructural parallels. This integrative view broadens classical endocrine anatomy and encourages further exploration of the immuno-endocrine microenvironment shared by these two closely related glands.

Very recent literature has revisited the versatility of pharyngeal endodermal programs and the functional “neighborhood” shared by these organs, reinforcing the concept of a morphologic and molecular continuum of communication that may become particularly relevant under specific conditions [35].

Within this framework, the IHC profile we have synthesized (Thymosin: high in the thymus, apical/heterogeneous in the parathyroid; CaSR: strong membranous/cytoplasmic expression in the parathyroid, weak and diffuse in the thymus; PTH1R: low–moderate in the thymus, moderate/heterogeneous in the parathyroid) aligns either partially or fully with the existing literature—fully in the case of CaSR in the parathyroid, partially for PTH1R, where extra-osseous evidence remains sporadic though biologically plausible, and in an original manner for Thymosin distribution in the parathyroid, where our data contribute a novel nuance of receptor topography.

Our current findings experimentally validate several of the theoretical premises proposed in our earlier review [6] confirming that the thymus–parathyroid interface is not only embryological but may also persist as a morpho-functional bridge in adult human tissues.

### Strengths and Limitations

A key strength of the study lies in its multi-scale coherence, combining IHC and SEM data, as well as the consistency observed across the different cohorts. Limitations include the absence of comprehensive IHC scoring suitable for advanced correlative analyses (which are planned for subsequent work) and the scarcity of literature specifically addressing thymosin expression in the parathyroid. This gap necessitated partial reliance on broader physiological and immunological sources rather than strictly parathyroid-focused IHC studies. Nonetheless, the concordance with established CaSR biology and the shared embryological origin of the parathyroid and thymus strengthens the mechanistic plausibility of a parathyroid–thymus axis.

Although the tissues were obtained from patients with oncologic pathology, all samples were excised from regions defined as oncologically safe, maintaining a minimum margin of 5–7 mm for parathyroid adenomas and at least 10 mm for thymic or mediastinal tumors. Each fragment was histologically verified to confirm the absence of neoplastic infiltration or inflammatory changes, in full compliance with oncologic safety standards.

## 4. Materials and Methods

### 4.1. Study Design

This manuscript presents a retrospective study based on archived surgical specimens. Tissue samples had been processed as part of routine histopathology and subsequently retrieved from paraffin blocks for further analysis. The study design ensured that all material was handled according to ethical requirements, with anonymisation of patient data and informed consent obtained whenever patients were recalled for additional investigations.

### 4.2. Participants

Three groups were included:

1. Thymus (*n* = 27): specimens obtained from thymectomies performed for thymoma. Sampling for this study was restricted to areas of macroscopically normal thymus, taken at least 5 mm away from the tumour margin, in order to respect oncological safety.
2. Parathyroid adenomas (*n* = 30): specimens excised for primary parathyroid adenoma. Only adenomatous tissue with histologically confirmed free surgical margins was included.
3. Parathyroids associated with thyroidectomy (*n* = 28): glands removed either incidentally or deliberately during total or subtotal thyroidectomy. Only fragments without gross pathological changes were selected.

In all cases, paraffin-embedded blocks were available for re-sectioning and immunohistochemical staining. Patients were also recalled for blood tests to evaluate the function of the non-operated glands, although these results are reported separately.

### 4.3. Biochemical and Hormonal Assessment

For all patients included in the three groups, serum biochemical parameters were retrospectively retrieved from institutional electronic records and, when available, confirmed through follow-up analyses performed within three months after surgery. The following laboratory markers were included:

- Ionic calcium (Ca^2+^) was determined by ion-selective electrode method (Roche Cobas 8000 analyzer, Roche Diagnostics, Mannheim, Germany), with a reference range of 3.8–4.8 mg/dL;
- Parathyroid hormone (PTH) was quantified using a second-generation electrochemiluminescence immunoassay (ECLIA) with normal values between 15 and 65 pg/mL;
- Thyroid-stimulating hormone (TSH) was measured by chemiluminescent microparticle immunoassay (CMIA) with reference interval 0.4–4.5 µIU/mL;
- Calcitonin was analyzed using immunoradiometric assay (IRMA) with reference limits < 10 pg/mL for females and <15 pg/mL for males;
- Anti-acetylcholine receptor (anti-AChR) antibodies was determined by radioimmunoassay (RIA) with a diagnostic threshold of 0.5 nmol/L.

All measurements were performed in duplicate under standard internal quality control procedures. Samples showing hemolysis or inadequate storage conditions were excluded from analysis. The results were expressed as mean ± standard deviation and correlated with histological findings to assess residual glandular function and systemic calcium–PTH homeostasis. It should be noted that the present study evaluated PTH1R and PTH at the protein ex-pression level by immunohistochemistry. No RNA transcriptional analysis was per-formed, and therefore, the results reflect protein distribution rather than gene expression patterns.

### 4.4. Histological and Immunohistochemical Analysis

Serial sections of 4 μm were cut from paraffin blocks. After standard deparaffinisation and rehydration, slides were stained with haematoxylin and eosin. Haematoxylin was applied for 5 min, followed by differentiation in acid alcohol, running tap water and eosin for 2–3 min. Sections were dehydrated, cleared in xylene and mounted permanently. Microscopic evaluation focused on thymic cortex and medulla, Hassall’s corpuscles, stromal organization, fibrous capsules and the distribution of chief and oxyphil cells in parathyroid tissue.

Consecutive 4-μm sections underwent deparaffinisation, rehydration and heat-induced epitope retrieval. Antigen retrieval was performed in EDTA buffer (pH 9.0) for PTH1R, Thymosin-α1 and CaSR; citrate buffer (pH 6.0) was used for Thymosin-β4 (unless otherwise specified). Endogenous peroxidase was blocked with 3% hydrogen peroxide and non-specific binding with normal serum. Slides were incubated overnight at 4 °C with the following primary antibodies and dilutions: PTH1R (1:100), Thymosin-α1 (1:100), CaSR (1:5000) and Thymosin-β4 (1:200). After PBS washes, HRP-conjugated secondary antibodies were applied for 30 min, chromogenic development was achieved with DAB, and counterstaining with haematoxylin was performed. Positive/negative controls were included in each run. Staining was assessed by localisation (cytoplasmic, membranous, nuclear) and by intensity (0–3+); the proportion of positive cells was recorded and H-score calculated for each case.

Incubation with HRP-conjugated secondary antibodies followed for 30 min. Colour development was achieved with diaminobenzidine, and counterstaining was performed with haematoxylin. Positive and negative controls were included in every series. Staining was assessed by localisation (cytoplasmic, membranous, or nuclear) and by intensity on a scale from 0 (absent) to 3+ (strong). The proportion of positive cells was recorded and an H-score was calculated for each case.

Immunohistochemical analysis was performed on formalin-fixed, paraf-fin-embedded breast carcinoma sections using the EnVision detection system (Dako, Glostrup, Denmark). The panel of antibodies included estrogen receptor (ER, clone 1D5, Dako, dilution 1:100), progesterone receptor (PR, clone PgR636, Dako, 1:100), HER2/neu (pol-yclonal, Dako, 1:500), Ki-67 (clone MIB-1, Dako, 1:200), p53 (clone DO-7, Dako, 1:100), and Thymosin β10 (TMSB10) (rabbit polyclonal antibody, Abcam, ab14335, 1:200, manufactured by Abcam Ltd., based in Cambridge, UK).

Antigen retrieval was performed by heat-induced epitope retrieval in citrate buffer (pH 6.0) for 20 min. Sections were incubated with primary antibodies for 60 min at room temperature, followed by HRP-conjugated secondary antibody and visualization with 3,3′-diaminobenzidine (DAB). Nuclear and/or cytoplasmic staining patterns were evaluated semiquantitatively according to the proportion of positive cells and the H-score method. Negative controls were obtained by omitting the primary antibody.

Quantitative immunohistochemical assessment.

The analysis of immunohistochemical markers was complemented by quantitative evaluation based on both the proportion of positive tumor cells and the H-score. Estrogen receptor (ER) expression showed a higher mean proportion of positive cells in the younger cohort (<40 years) compared to the older group (≥40 years), with corresponding mean H-scores of 246 ± 34 and 198 ± 39, respectively (*p* = 0.021). Progesterone receptor (PR) levels followed a similar pattern (H-score 218 ± 37 vs. 184 ± 41, *p* = 0.034).

In contrast, HER2 expression and Ki-67 proliferative index were significantly higher in tumors from younger patients (H-score 156 ± 47 vs. 118 ± 35 for HER2, *p* = 0.042; mean Ki-67 positivity 39.5% ± 11.2 vs. 26.7% ± 8.9, *p* = 0.008), supporting a more aggressive phenotype. The quantitative data for all immunomarkers, including standard deviations and *p*-values, are summarized in Supplementary Table S1.

### 4.5. Scanning Electron Microscopy (SEM) Analysis

Selected fragments of thymus and parathyroid tissue were processed for SEM following established protocols. The analysis focused on the three-dimensional architecture of the stroma, the fibrous capsule, vascular arrangements and surface specializations of epithelial and parenchymal cells.

The ultrastructural component of the study was carried out on the same specimens used for histological and immunohistochemical analyses. To achieve high-resolution imaging, samples were processed and examined under high-vacuum conditions in a scanning electron microscope.

Fresh tissue fragments were initially fixed in 2.5% glutaraldehyde, buffered with 0.1 M sodium cacodylate (pH 7.4), and subsequently post-fixed in 1% osmium tetroxide. Dehydration was performed stepwise in graded ethanol solutions (30%, 50%, 70%, 90%, 100%; 10 min each). Critical point drying with carbon dioxide ensured optimal preservation of morphology. Specimens were then mounted on aluminum stubs and coated with a thin layer of gold (approximately 7–10 nm) to provide conductivity for electron beam exposure.

Observations were performed with a VegaTescan LMH II scanning electron microscope (Brno-Kohoutovice, Czech Republic), equipped with a tungsten filament, a secondary electron detector, and software for high-resolution imaging. The instrument allowed magnifications up to 100,000×, providing detailed surface relief and structural organization of the tissues.

In addition to conventional tissue fragments, selected paraffin-embedded sections stained with haematoxylin and eosin were adapted for ultrastructural examination. For this purpose, slides were carefully demounted using graded alcohol and xylene solutions to remove the coverslip and resin-based mounting medium without compromising the integrity of the stained section. Recovered tissues were rehydrated, post-fixed in osmium tetroxide, dehydrated once more, and prepared for sputter-coating and SEM analysis. This approach preserved the architecture of the original histological section while adding a complementary three-dimensional ultrastructural perspective.

Examination was conducted under medium vacuum at accelerating voltages between 5 and 15 kV, with both secondary electrons (for topographic detail) and backscattered electrons (for compositional contrast). This methodology enabled the assessment of fine surface details, including the organization of the extracellular matrix, vascular structures, and age- or pathology-related alterations of tissue microarchitecture.

### 4.6. Ethical Considerations

The study was approved by the institutional ethics committee of Grigore T. Popa University of Medicine and Pharmacy Iasi, Romania, number 382 from 18 January 2024. and complied with the principles of the Declaration of Helsinki (2013). All tissue was anonymized. Patients who were recalled for blood sampling provided written informed consent.

### 4.7. Statistical Analysis

Quantitative and semi-quantitative variables derived from immunohistochemical scoring and SEM evaluation were analyzed using non-parametric statistics. Normality of distribution was tested by the Shapiro–Wilk method. Inter-group comparisons among the three study cohorts were performed with the Kruskal–Wallis test, followed by Dunn’s post hoc analysis with Benjamini–Hochberg false discovery rate (FDR) adjustment. Correlations between IHC marker expression (Thymosin-α1, Thymosin-β4, PTH1R, and CaSR) and ultrastructural indices (Vascular Surface Index, Ultrastructural Integrity Score, and Composite Structural Score) were evaluated using Spearman’s rank correlation coefficients.

An ordinal logistic regression model was fitted to identify independent predictors of structural organization, with the Composite Structural Score (CSS) as the dependent variable and IHC markers as covariates, adjusting for age and cohort type. Inter-observer reliability for IHC scoring and SEM grading was assessed using Cohen’s weighted kappa coefficient. Statistical significance was defined as *p* < 0.05 (two-tailed). All analyses were conducted using SPSS Statistics version 26.0 (IBM Corp., Armonk, NY, USA) and GraphPad Prism version 9.0 (GraphPad Software, San Diego, CA, USA).

## 5. Conclusions

This study gives converging ultrastructural and immunohistochemical evidence supporting a functional link between the thymus and parathyroid. Thymosin, CaSR, and PTH1R display differentiated yet complementary expression patterns, with CaSR confirming canonical parathyroid specificity, PTH1R extending evidence for extra-osseous roles, and Thymosin distribution in the parathyroid emerging as a novel finding. Together, these results reinforce the concept that beyond their common embryological origin, the two glands may sustain a morpho-molecular continuum with potential clinical implications.

## Data Availability

The original contributions presented in this study are included in the article and Supplementary Materials. Further inquiries can be directed to the corresponding author.

## Supplementary Materials

The following supporting information can be downloaded at: https://www.mdpi.com/article/10.3390/ijms262311561/s1.

## References

[1] Scharpf J., Kyriazidis N., Kamani D., Randolph G. (2016). Anatomy and embryology of the parathyroid gland. Oper. Tech. Otolaryngol.-Head Neck Surg..

[2] Rosen R.D., Bordoni B. (2025). Embryology, Parathyroid. StatPearls.

[3] Comanescu M.P., Boisteanu O., Hînganu D., Hînganu M.V., Grigorovici R., Grigorovici A. (2025). Perspectives on the Parathyroid-Thymus Interconnection-A Literature Review. Int. J. Mol. Sci..

[4] Alcobia I., Gama-Carvalho M., Magalhães L., Proa V., Ferreira S., Henrique D., Neves H. (2024). Thymus-forming potential of the second pharyngeal pouch and its regulation by local mesenchyme in avian embryos. Cell Rep..

[5] Cuciureanu D.I., Statescu C., Sascau R.A., Cuciureanu T., Constantinescu V.A., Hinganu D., Preda C., Hinganu M.V., Turliuc M.D. (2019). Particularities of Using Contrast Agents in Diagnosis of Stroke. Rev. Chim..

[6] Comanescu M.P., Boisteanu O., Sulea D., Frij-Calin A.I., Patrascanu E., Grigorovici R., Hînganu M.V., Hînganu D., Grigorovici A. (2024). Comparative study between postoperative parathyroid deficiency hypocalcemia in those with reimplantation versus those without reimplantation. Rom. J. Oral Rehabil..

[7] Denkboy Öngen Y., Özemri Sağ Ş., Temel Ş.G., Eren E. (2023). An Endocrinological Perspective on 22q11.2 Deletion Syndrome: A Single-center Experience. J. Clin. Res. Pediatr. Endocrinol..

[8] Mustillo P.J., Sullivan K.E., Chinn I.K., Notarangelo L.D., Haddad E., Davies E.G., de la Morena M.T., Hartog N., Yu J.E., Hernandez-Trujillo V.P. (2023). Clinical Practice Guidelines for the Immunological Management of Chromosome 22q11.2 Deletion Syndrome and Other Defects in Thymic Development. J. Clin. Immunol..

[9] McDonald-McGinn D.M., Hain H.S., Emanuel B.S., Zackai E.H., Adam M.P., Feldman J., Mirzaa G.M., Pagon R.A., Wallace S.E., Amemiya A. (1993). 22q11.2 Deletion Syndrome. GeneReviews^®^.

[10] Scheuerle A.E., Geleske T.A., Merchant N., Goldenberg P.C. (2025). Health Supervision for Children With 22q11.2 Deletion Syndrome: Clinical Report. Pediatrics.

[11] Vaidya H.J., Briones Leon A., Blackburn C.C. (2016). FOXN1 in thymus organogenesis and development. Eur. J. Immunol..

[12] Bosticardo M., Notarangelo L.D. (2023). Human thymus in health and disease: Recent advances in diagnosis and biology. Semin. Immunol..

[13] Zhao J., Hu R., Lai K.C., Zhang Z., Lai L. (2024). Recombinant FOXN1 fusion protein increases T cell generation in old mice. Front. Immunol..

[14] Nagakubo D., Hirakawa M., Iwanami N., Boehm T. (2022). Limits to in vivo fate changes of epithelia in thymus and parathyroid by ectopic expression of transcription factors Gcm2 and Foxn1. Sci. Rep..

[15] Salahoru P., Grigorescu C., Hinganu M.V., Lunguleac T., Halip A.I., Hinganu D. (2024). Thymus Surgery Prospectives and Perspectives in Myasthenia Gravis. J. Pers. Med..

[16] Geara A.S., Castellanos M.R., Bassil C., Schuller-Levis G., Park E., Smith M., Goldman M., Elsayegh S. (2010). Effects of parathyroid hormone on immune function. Clin. Dev. Immunol..

[17] Yu M., Malik Tyagi A., Li J.Y., Adams J., Denning T.L., Weitzmann M.N., Jones R.M., Pacifici R. (2020). PTH induces bone loss via microbial-dependent expansion of intestinal TNF(+) T cells and Th17 cells. Nat. Commun..

[18] Garaci E., Paci M., Matteucci C., Costantini C., Puccetti P., Romani L. (2024). Phenotypic drug discovery: A case for thymosin alpha-1. Front. Med..

[19] Besman M., Zambrowicz A., Matwiejczyk M. (2024). Review of Thymic Peptides and Hormones: From Their Properties to Clinical Application. Int. J. Pept. Res. Ther..

[20] Kunstek H., Kieviet J., Lindemans C., de Koning C., Nierkens S. (2025). Thymic peptides in immune reconstitution and clinical outcome after allogeneic hematopoietic cell transplantation. Blood Neoplasia.

[21] Liu Z., Farley A., Chen L., Kirby B.J., Kovacs C.S., Blackburn C.C., Manley N.R. (2010). Thymus-associated parathyroid hormone has two cellular origins with distinct endocrine and immunological functions. PLoS Genet..

[22] Michelson D.A., Mathis D. (2024). Thymic Mimetic Cells: Ontogeny as Immunology. Annu. Rev. Cell. Dev. Biol..

[23] Bhalla P., Su D.M., van Oers N.S.C. (2022). Thymus Functionality Needs More Than a Few TECs. Front. Immunol..

[24] Şenkal-Turhan S., Bulut-Okumuş E., Aydın M., Başak Türkmen N., Taşlıdere A., Şahin F., Yılmaz Ş., Akkuş Süt P., Doğan A. (2024). Induced Pluripotent Stem Cell-Derived Parathyroid Organoids Resemble Parathyroid Morphology and Function. Adv. Sci..

[25] Tao N., Xu X., Ying Y., Hu S., Sun Q., Lv G., Gao J. (2023). Thymosin α1 and Its Role in Viral Infectious Diseases: The Mechanism and Clinical Application. Molecules.

[26] Li X., Lu Y., Zhang L., Song A., Zhang H., Pang B., Liu J., Sun X., Ji H., Huang L. (2023). Primary and secondary hyperparathyroidism present different expressions of calcium-sensing receptor. BMC Surg..

[27] Worth A.L., Ayrapetyan M., Maygarden S.J., Li Z., Wu Z., Agala C.B., Kim L.T. (2024). Expression of the Calcium-Sensing Receptor on Normal and Abnormal Parathyroid and Thyroid Tissue. J. Surg. Res..

[28] Yuan M., Ma T., Fan Z., Li J., Zhang S. (2025). The calcium-sensing receptor: A comprehensive review on its role in calcium homeostasis and therapeutic implications. Am. J. Transl. Res..

[29] Sjöstedt E., Zhong W., Fagerberg L., Karlsson M., Mitsios N., Adori C., Oksvold P., Edfors F., Limiszewska A., Hikmet F. (2020). An atlas of the protein-coding genes in the human, pig, and mouse brain. Science.

[30] Lyu P., Li B., Li P., Bi R., Cui C., Zhao Z., Zhou X., Fan Y. (2021). Parathyroid Hormone 1 Receptor Signaling in Dental Mesenchymal Stem Cells: Basic and Clinical Implications. Front. Cell Dev. Biol..

[31] Zhai X., Mao C., Shen Q., Zang S., Shen D.-D., Zhang H., Chen Z., Wang G., Zhang C., Zhang Y. (2022). Molecular insights into the distinct signaling duration for the peptide-induced PTH1R activation. Nat. Commun..

[32] Karabiyik Acar Ö., Nozhatzadeh G.D., Tuncer A., Torun Köse G., Hacihasanoğlu E., Sahin F., Aysan E. (2022). Production of parathyroid-like cells from thyroid stem cells in co-culture environment. Medicine.

[33] Kim S., Shin J.H., Hahn S.Y., Kim H., Kim M.K. (2024). The Parathyroid Gland: An Overall Review of the Hidden Organ for Radiologists. J. Korean Soc. Radiol..

[34] Xu Q., La T., Ye K., Wang L., Wang S., Hu Y., Teng L., Yan L., Li J., Zhang Z. (2024). KMT2A and chronic inflammation as potential drivers of sporadic parathyroid adenoma. Clin. Transl. Med..

[35] Figueiredo M., Zilhão R., Neves H. (2020). Thymus Inception: Molecular Network in the Early Stages of Thymus Organogenesis. Int. J. Mol. Sci..

