# Supplementary material for: Cross-Expression of Thymic and Parathyroid Hormone Receptors Supports the Hypothesis of a Parathyroid–Thymus Port System
Source: Int J Mol Sci. 2025 Nov 28;26(23):11561. doi: 10.3390/ijms262311561 (PMC12691803; doi:10.3390/ijms262311561)
Supplement: Supplementary file 1 [file ijms-26-11561-s001.zip › ijms-3970275-supplementary.pdf]

Table S1. Individual demographic and biochemical characteristics of donors with thymic tissue samples (patients diagnosed with thymoma).

Pacient	Sex	Age	Ionic Ca (mg/dl)	TSH (μUI/mL)	PTH (pg/mL)	Calcitonine (pg/mL)	Anti-AchR (nmol/L)
P1	F	56	4.491	4.49	62.01	2.24	0.009
P2	F	37	4.152	2.84	68.74	2.04	0.010
P3	M	42	4.01	3.22	103.63	4.12	15.20
P4	F	53	3.974	2.90	70.54	2.10	0.014
P5	M	26	4.273	2.71	65.32	2.30	3.64
P6	F	37	4.14	2.94	73.20	2.41	16.31
P7	F	42	3.764	4.79	59.00	2.01	7.54
P8	F	41	4.109	3.38	71.81	2.42	6.088
P9	M	41	4.111	3.42	71.81	2.43	6.090
P10	F	41	4.110	3.44	71.84	2.43	6.087
P11	M	41	4.108	3.40	71.76	2.41	6.087
P12	F	41	4.110	3.42	71.76	2.39	6.086
P13	M	41	4.107	3.36	71.79	2.36	6.088
P14	F	41	4.107	3.37	71.78	2.37	6.092
P15	M	41	4.109	3.37	71.77	2.38	6.091
P16	F	41	4.106	3.39	71.79	2.37	6.090
P17	M	41	4.110	3.38	71.80	2.38	6.090
P18	F	41	4.112	3.40	71.82	2.39	6.090
P19	M	41	4.111	3.40	71.76	2.44	6.092
P20	F	41	4.108	3.41	71.76	2.44	6.087
P21	M	41	4.114	3.43	71.79	2.43	6.087
P22	F	41	4.109	3.42	71.78	2.41	6.086
P23	M	41	4.114	3.40	71.80	2.40	6.089
P24	F	41	4.112	3.44	71.80	2.42	6.094
P25	M	41	4.110	3.36	71.81	2.41	6.093

Table S2. Individual demographic and biochemical characteristics of donors with parathyroid tissue samples (patients diagnosed with parathyroid adenoma).

Patient	Sex	Age	Ionic Ca (mg/dL)	TSH (μUI/mL)	PTH (pg/mL)	Calcitonin (pg/mL)	Anti-AchR (nmol/L)
P1	F	25	8.72	3.08	98.19	2.17	0.06
P2	M	32	8.75	3.67	92.25	2.26	0.03
P3	F	35	8.36	3.4	90.34	2.16	0.06
P4	M	38	8.64	3.21	108.12	2.44	0.03
P5	F	40	8.86	3.53	100.25	2.14	0.07
P6	M	42	8.46	3.12	92.56	2.46	0.06
P7	F	44	8.86	3.56	106.44	2.52	0.04
P8	M	47	8.64	3.62	109.04	2.47	0.05
P9	F	49	8.78	3.09	96.7	2.2	0.03
P10	M	28	8.37	3.13	98.29	2.36	0.04
P11	F	30	8.49	3.2	97.94	2.5	0.06

P12	M	33	8.69	3.18	112.54	2.15	0.04
P13	F	36	9.02	3.5	107.1	2.38	0.07
P14	M	39	8.84	3.22	90.52	2.22	0.04
P15	F	41	8.35	3.7	117.19	2.21	0.06
P16	M	43	8.51	3.68	104.0	2.19	0.04
P17	F	45	8.65	3.24	107.97	2.48	0.04
P18	M	48	8.36	3.74	105.6	2.11	0.03
P19	F	50	8.26	3.49	114.53	2.26	0.03
P20	M	29	8.5	3.74	106.22	2.52	0.07
P21	F	31	8.18	3.55	111.04	2.32	0.04
P22	M	34	8.72	3.14	103.24	2.28	0.07
P23	F	37	8.92	3.24	105.32	2.15	0.07
P24	M	46	8.92	3.26	109.69	2.35	0.03
P25	F	52	8.83	3.43	114.1	2.31	0.03
P26	M	54	8.45	3.07	118.86	2.47	0.07
P27	F	27	8.43	3.1	117.24	2.51	0.03
P28	M	42	8.59	3.11	113.53	2.42	0.03

Table S3. Individual demographic and biochemical characteristics of donors who underwent subtotal thyroidectomy (control group).

**Patient**	**Sex**	**Age**	**Ionic Ca (mg/dL)**	**TSH (μUI/mL)**	**PTH (pg/mL)**	**Calcitonin (pg/mL)**	**Anti-AChR (nmol/L)**
P1	F	29	4.63	3.5	140.4	2.24	0.005
P2	M	33	4.69	3.14	130.7	2.28	0.007
P3	F	36	4.9	3.35	150.6	2.51	0.014
P4	M	38	4.64	3.2	149.5	2.29	0.01
P5	F	40	4.66	3.24	167.8	2.33	0.015
P6	M	42	4.71	3.72	153.1	2.47	0.009
P7	F	44	4.48	3.61	158.6	2.15	0.006
P8	M	47	4.67	3.27	152.2	2.14	0.011
P9	F	53	4.73	3.66	159.6	2.49	0.013
P10	M	57	4.82	3.2	150.3	2.45	0.008
P11	F	49	4.43	3.33	142.1	2.18	0.006
P12	M	31	4.55	3.64	174.9	2.16	0.008
P13	F	35	4.43	3.5	174.8	2.41	0.015
P14	M	41	4.83	3.13	167.7	2.5	0.013
P15	F	45	4.77	3.73	161.7	2.16	0.006
P16	M	50	4.4	3.21	143.8	2.51	0.007
P17	F	28	4.93	3.24	139.9	2.48	0.006
P18	M	34	4.92	3.59	142.6	2.35	0.006
P19	F	37	4.75	3.28	132.6	2.26	0.013

P20	M	39	4.72	3.26	164.4	2.12	0.007
P21	F	43	4.47	3.11	147.7	2.09	0.011
P22	M	46	4.39	3.12	168.0	2.51	0.009
P23	F	52	4.68	3.46	147.0	2.18	0.007
P24	M	55	4.41	3.23	173.1	2.39	0.012
P25	F	30	4.49	3.47	168.1	2.19	0.006
P26	M	32	5.06	4.21	96.0	1.96	0.03

Table S4. Quantitative immunohistochemical assessment (percentage of positive cells and H-scores) according to age group

Marker	Age Group	% Positive Cells (Mean ± SD)	H-score (Mean ± SD)	p-value
ER	< 40 years	82.4 ± 10.2	246 ± 34	0.021
	≥ 40 years	69.7 ± 12.4	198 ± 39	
PR	< 40 years	77.3 ± 13.5	218 ± 37	0.034
	≥ 40 years	63.8 ± 15.1	184 ± 41	
HER2	< 40 years	45.1 ± 19.2	156 ± 47	0.042
	≥ 40 years	32.6 ± 16.5	118 ± 35	
Ki-67	< 40 years	39.5 ± 11.2	—	0.008
	≥ 40 years	26.7 ± 8.9	—	
p53	< 40 years	33.4 ± 10.7	122 ± 28	0.067
	≥ 40 years	27.1 ± 9.3	111 ± 25	

Data are presented as mean ± standard deviation. Statistical significance was determined using Student's t-test or Mann–Whitney U test as appropriate.

Table S5. Thymosin β10 (TMSB10) immunohistochemistry in adult parathyroid tissues

Lesion type	n	Cell type	% Positive cells (Mean ± SD)	H-score (Mean ± SD)
Normal parathyroid	10	Chief	27.1 ± 8.4	94 ± 32
Normal parathyroid	10	Oxyphil	16.2 ± 6.5	71 ± 26
Parathyroid adenoma	24	Chief	44.8 ± 12.3	172 ± 41
Parathyroid adenoma	24	Oxyphil	29.7 ± 10.2	111 ± 29
Parathyroid hyperplasia	14	Chief	34.7 ± 10.9	138 ± 38
Parathyroid hyperplasia	14	Oxyphil	21.9 ± 8.5	94 ± 27

Data are expressed as mean ± standard deviation. Statistical significance was determined by ANOVA followed by Tukey's post hoc test.

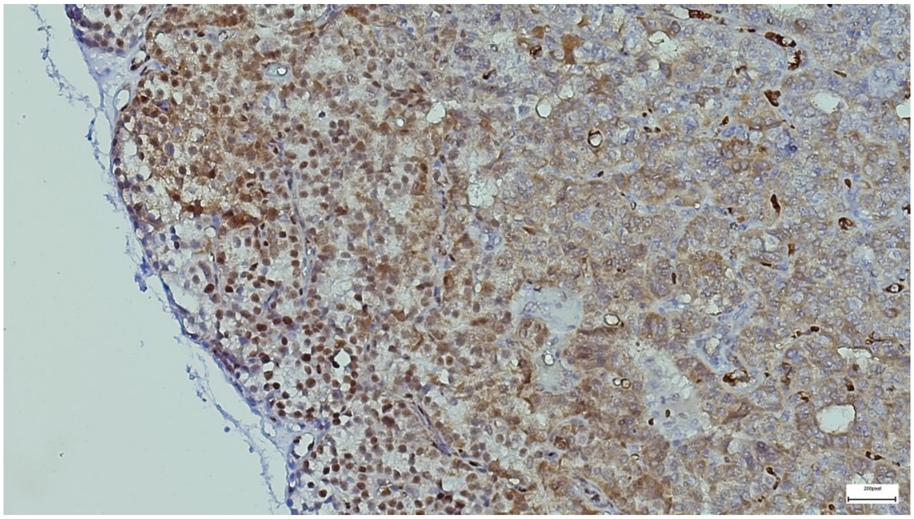

Figure S1 Parathoroid: Thymosin expression (Scale bar = 200 pixel).

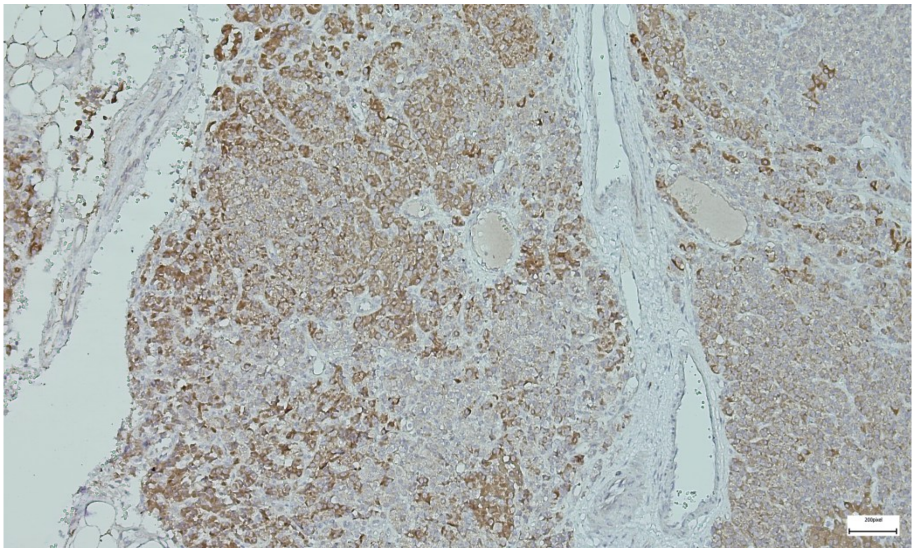

Figure S2 Parathoroid: PTH1R heterogeny expression (Scale bar = 200 pixel).

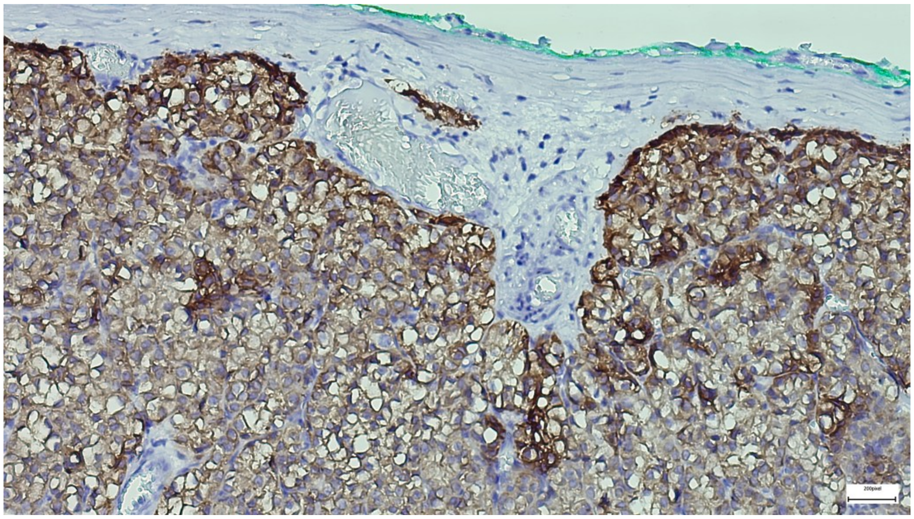

Figure S3 Parathoroid: CaSR (Scale bar = 200 pixel).

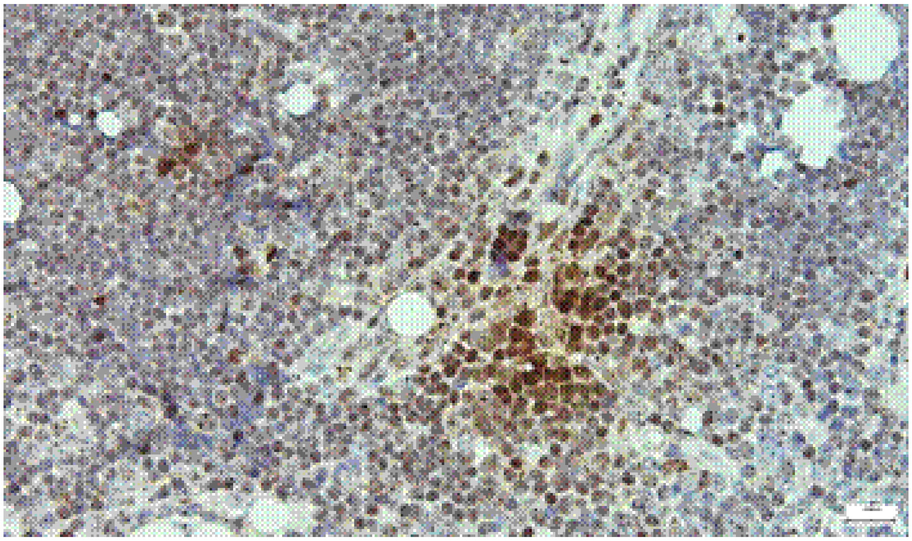

Figure S4 Thymus: tymosin expression in thymocytes (Scale bar = 200 pixel).

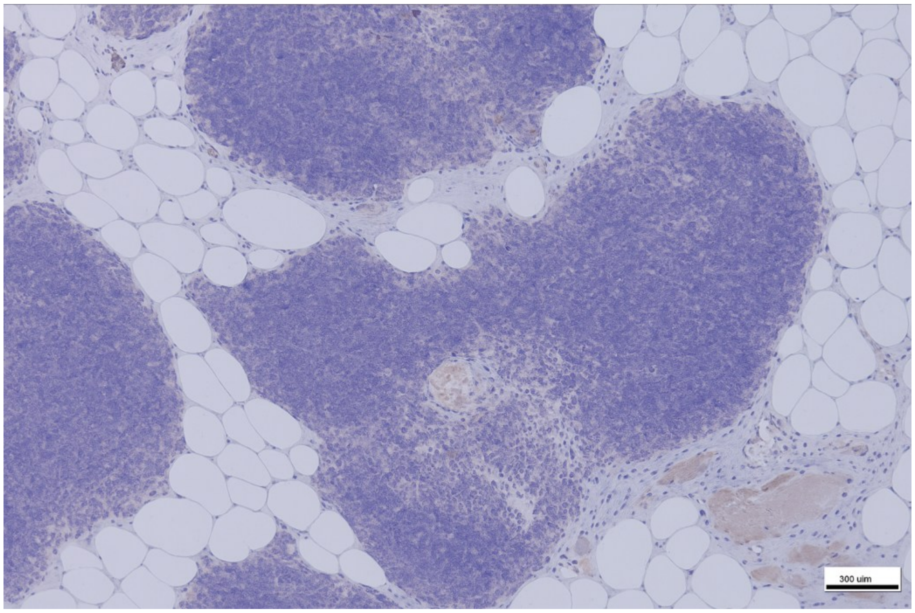

Figure S5 Thymus: PTH1R showed weak, focal cytoplasmic or membranous staining

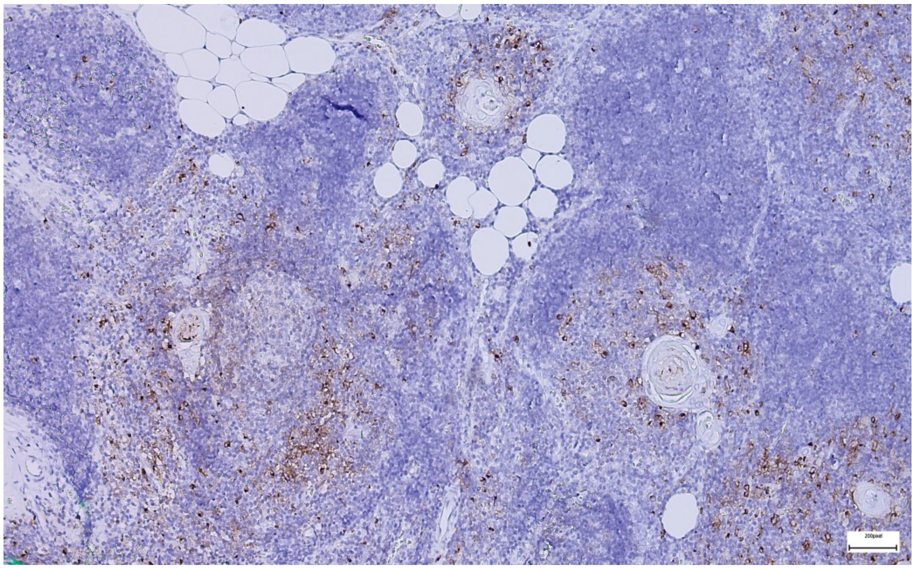

Figure S6 Thymus: CaSR (Scale bar = 200 pixel).

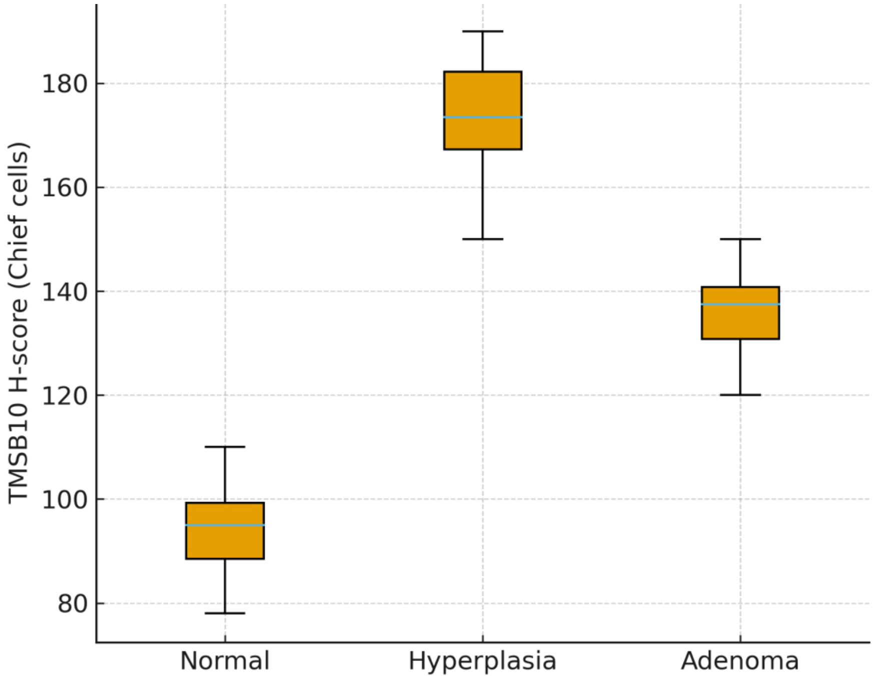

Figure S7. Thymosin β10 (TMSB10) Comparative box-plot illustrating Thymosin β10 (TMSB10) H-scores in chief cells from normal parathyroid tissue, parathyroid hyperplasia, and parathyroid adenoma. A progressive increase in TMSB10 expression is observed from normal glands to hyperplasia and adenoma ($p < 0.001$, ANOVA). Data are expressed as median, interquartile range, and range.
